# Image Quality and Interpretation of [^18^F]-FES-PET: Is There any Effect of Food Intake?

**DOI:** 10.3390/diagnostics10100756

**Published:** 2020-09-26

**Authors:** Jorianne Boers, Katerina Giatagana, Carolina P. Schröder, Geke A.P. Hospers, Erik F.J. de Vries, Andor W.J.M. Glaudemans

**Affiliations:** 1 Department of Medical Oncology, University Medical Center Groningen, University of Groningen, 9700 RB Groningen, The Netherlands; a.giatagana@student.rug.nl (K.G.); c.p.schroder@umcg.nl (C.P.S.); g.a.p.hospers@umcg.nl (G.A.P.H.); 2 Department of Nuclear Medicine and Molecular Imaging, University Medical Center Groningen, University of Groningen, 9700 RB Groningen, The Netherlands; e.f.j.de.vries@umcg.nl (E.F.J.d.V.); a.w.j.m.glaudemans@umcg.nl (A.W.J.M.G.)

**Keywords:** FES-PET, breast cancer, abdominal distribution, fasting, chocolate

## Abstract

Background: High physiological 16α-[^18^F]-fluoro-17β-estradiol ([^18^F]-FES) uptake in the abdomen is a limitation of this positron emission tomography (PET) tracer. Therefore, we investigated the effect of food intake prior to PET acquisition on abdominal background activity in [^18^F]-FES-PET scans. Methods: Breast cancer patients referred for [^18^F]-FES-PET were included. Three groups were designed: (1) patients who consumed a chocolate bar (fatty meal) between tracer injection and imaging (*n* = 20), (2) patients who fasted before imaging (*n* = 20), and (3) patients without diet restrictions (control group, *n* = 20). We compared the physiological [^18^F]-FES uptake, expressed as mean standardized uptake value (SUV_mean_), in the abdomen between groups. Results: A significant difference in [^18^F]-FES uptake in the gall bladder and stomach lumen was observed between groups, with the lowest values for the chocolate group and highest for the fasting group (*p* = 0.015 and *p* = 0.011, respectively). Post hoc analysis showed significant differences in the SUVmean of these organs between the chocolate and fasting groups, but not between the chocolate and control groups. Conclusion: This exploratory study showed that, compared to fasting, eating chocolate decreases physiological gall bladder and stomach [^18^F]-FES uptake; further reduction through a normal diet was not seen. A prospective study is warranted to confirm this finding.

## 1. Introduction

Information about estrogen receptor (ER) expression in breast cancer (BC) is essential, because it can guide important therapy decisions. ER-positive lesions are found in about 70% of BCs, and these tumors are likely to respond to endocrine treatment [1]. Nowadays, the gold standard for determining the ER status is immunohistochemistry of biopsy samples. However, in clinical practice, a biopsy is not always feasible or may lead to sample errors. When diagnostic dilemmas cannot be solved with conventional imaging methods, molecular imaging with 16α-[^18^F]-fluoro-17β-estradiol ([^18^F]-FES) positron emission tomography (PET) may be used to obtain, in a non-invasive way, whole-body information about ER expression of all tumor lesions within the body. This imaging technique may improve diagnostic understanding and may help in therapy decision-making [2,3]. However, there are limitations in the use of this molecular imaging technique, such as high physiological [^18^F]-FES uptake in the gall bladder and bile ducts, and excretion into the gastrointestinal tract. [^18^F]-FES is rapidly metabolized in the liver and excreted by the gall bladder and bile ducts into the gastrointestinal tract, resulting in high background activity in these organs, which can hamper interpretation of [^18^F]-FES-PET in the abdominal region. This limitation of [^18^F]-FES-PET is especially important in patients with ovarian tumors, which most commonly develop intra-abdominal metastases [4].

Methods to enhance the contrast between ER-positive tumor lesions and physiological uptake could aid the accurate visualization of abdominal metastases. One method could be to increase the hepatobiliary clearance rate of the tracer. This might potentially be achieved by stimulating gall bladder contraction with a (fatty) meal around the time of tracer injection, which would result in faster passage of the tracer from the liver to duodenum. This method is commonly used in nuclear medicine for myocardial perfusion imaging [5,6,7,8,9]. Several high-fat meals have been investigated in the field of myocardial perfusion, such as milkshakes [6], full-fat milk [7,8], and chocolate [9]. Our hospital uses chocolate as a fatty meal between injection and myocardial perfusion imaging, and therefore chocolate was selected for this study as well. Chocolate has also been used as a high-lipid food in studies investigating cholecystography [10].

On the other hand, fasting (at least 4 h before imaging) has also been suggested to reduce bowel accumulation due to reduced elimination of [^18^F]-FES from the gall bladder [11]. Nowadays, different patient preparation instructions are used for [^18^F]-FES-PET imaging, such as fasting, oral hydration with water, and non-fasting [11,12,13]. The first recommendation paper about [^18^F]-FES-PET concluded that no specific patient preparation instructions are necessary, except when the PET scan is acquired in combination with a diagnostic (contrast-enhanced) computed tomography (CT)-scan [14].

The aim of this single-center exploratory study is to assess the effect of food intake prior to PET acquisition (a fatty meal (chocolate) between tracer injection and imaging, a period of fasting prior to imaging, and no diet restrictions) on abdominal background [^18^F]-FES activity in patients with BC. We hypothesized that the administration of chocolate before [^18^F]-FES-PET imaging would lead to faster excretion, resulting in less-disturbing physiological [^18^F]-FES uptake in the gall bladder and upper gastrointestinal tract, whereas fasting would increase background tracer uptake in these organs. 

## 2. Materials and Methods

### 2.1. Patients

We included BC patients who have been referred for [^18^F]-FES-PET by their treating physician as part of routine clinical practice in the University Medical Center Groningen (UMCG) from February 2012 till August 2019. [^18^F]-FES-PET was requested because of a clinical dilemma, when standard workup was inconclusive. Patients were divided into three groups based on their food intake before the PET scan:

(1) Chocolate group (used as a fatty meal), which included 20 prospectively collected, consecutive patients who ate a chocolate bar directly after tracer injection and before [^18^F]-FES-PET imaging. The weight of the milk chocolate bar was 47 g, and contained 248 kcal (1036 kJ). The amount of fat was 14 g, or 20% of total daily fat intake.
(2) Fasting group (*n* = 20), which included retrospectively collected patients without any food intake at least 4–6 h prior to the [^18^F]-FES-PET scan, and patients who were instructed to fast due to combined contrast-enhanced CT-scan.
(3) Control group without any diet restrictions prior to tracer injection (which was our standard patient preparation; *n* = 20). This group was also retrospectively selected from a larger database.

Patients were excluded when [^18^F]-FES-PET was performed for research purposes, if they had an intolerance to or refused to eat chocolate, and if liver metastases were present to avoid overlap of tracer uptake in tumors with the gall bladder and/or bile ducts. Both the fasting and control groups were matched based on sex, age, weight, menopausal status (postmenopausal status was defined as age ≥60 years, age <60 years and amenorrhea for >12 months without oral contraceptives, or chemical/surgical ovarian function suppression), and use of ER-antagonists (treatment was stopped at least 5 weeks before the PET scan), using the chocolate group as reference.

### 2.2. Study Design

This was a single-center exploratory study performed at the UMCG, the Netherlands. The Medical Ethics Committee of the UMCG has reviewed the protocol and decided that this type of research was beyond the scope of the Medical Research Involving Human Subjects Act (WMO) (METc 2018/2017). Patients in the chocolate group did provide verbal informed consent for consuming chocolate prior to the scan. The primary endpoint of the study was the physiological [^18^F]-FES uptake in the gastrointestinal tract (liver, gall bladder or bile ducts, small and large bowel), expressed as mean standardized uptake value (SUV_mean_). 

### 2.3. [^18^F]-FES-PET

Patients received ~200 MBq of [^18^F]-FES intravenously. Whole-body (head to mid-thigh) PET/CT was performed 60 min after tracer injection using a Siemens Biograph (Siemens Healthineers, Knoxville, TN, USA) 40- or 64-slice mCT with a PET emission acquisition time of 3 min per bed position. Low-dose CT was acquired for attenuation and scatter correction. Reconstructions of the scan and quantification were performed according to the European Association of Nuclear Medicine (EANM, Vienna, Austria) guidelines for ^18^F imaging and the EANM Research Limited (EARL, Vienna, Austria) criteria [15]. All quantifications were performed on EARL reconstructed images with a 2 mm reconstructed spatial resolution. 

### 2.4. [^18^F]-FES-PET Data Analysis

We used *syngo*.via VB30 imaging software (Siemens Healthineers, Knoxville, TN, USA) for quantitative measurements of tracer uptake in various abdominal organs. All PET images were evaluated for physiological [^18^F]-FES uptake by drawing volumes of interest (VOIs) in the lumen and wall of various parts of the gastrointestinal tract (the colon was dived into: the cecum, the ascending, transverse, and descending sections, and sigmoid, and rectum), liver, gall bladder, and the blood pool as reference. The median [^18^F]-FES uptake and interquartile range per organ and per group were reported. Tracer uptake in the liver was determined by placing 3 spherical VOIs with a diameter between 2.5 and 3.5 cm (segment 2, 5/8 and 6). The average tracer uptake in these VOIs was reported. Two measurements were performed for the stomach (wall and lumen) and the blood pool (aortic arch and descending thoracic aorta). For patients who underwent a gall bladder excision in the past, [^18^F]-FES uptake was determined in the bile ducts instead of the gall bladder. All measurements were performed by a trained observer (KG; unaware of the patient grouping), and doublechecked by a second trained observer (JB), all under supervision of an experienced nuclear medicine physician (AG). 

### 2.5. Statistical Analysis

Descriptive analysis of [^18^F]-FES uptake per organ and per group was performed. When the results were normally distributed, continuous variables were expressed as mean ± standard deviation (SD); otherwise, median and interquartile range were noted. Categorical variables were expressed as numbers (percentage) and analyzed with the Chi-square test. Continuous and normally distributed variables were analyzed with the One-Way ANOVA test. The Kruskal–Wallis test was used for comparisons between differences in physiological [^18^F]-FES uptake between groups, because the groups were unrelated and lacked normality (normal distribution of the data could not be proven with the Shapiro–Wilk test, Levene’s test and Q–Q plot). To test the significance of the differences between individual groups and correct for multiple comparisons, we used the Dunn–Bonferroni post hoc correction method. Statistical significance was defined by a probability (*p*) value ≤ 0.05. Analyses were performed using IBM SPSS Statistics for Windows, version 23 (IBM Corp., Armonk, NY, USA).

## 3. Results

### 3.1. Patients

Patient characteristics are summarized in Table 1. The majority of patients (78%) had metastatic disease. There were no statistically significant differences in age, weight, BMI, and menopausal status between the three groups. All patients who used ER-antagonists discontinued this treatment >5 weeks before [^18^F]-FES-PET imaging. All patients who received a chocolate bar ate the whole bar, except for one patient who ate 5/6 of the bar. Since the control group without diet restriction was established retrospectively, no information was available about their food consumption prior to the PET scan. However, based on the information that the tracer injection is always administered during the early afternoon, it is likely that patients had had lunch before the scan. Similarly, the exact number of fast hours is unknown in the fasting group. The majority of patients (16/20) needed to fast for at least 4–6 h prior to the [^18^F]-FES-PET scan, and 4 patients were instructed to fast due to combined contrast-enhanced CT-scan. Of these 4 patients, three patients probably had to fast for at least 3.5 h, and one patient for at least 2.5 h. 

### 3.2. Physiological [^18^F]-FES Uptake

**Figure 1:**
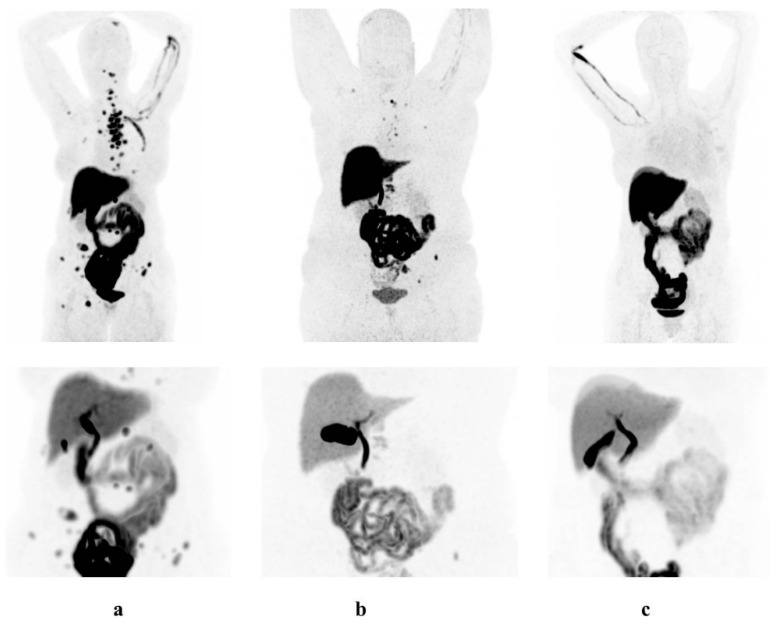
[^18^F]-FES-PET scans of breast cancer patients showing visual enhanced physiological [^18^F]-FES uptake in all protocols: (**a**) chocolate, (**b**) fasting, and (**c**) control group.

Table 2 and Figure 1 summarize the physiological [^18^F]-FES uptake in the abdomen for the three groups. On visual analysis, no clear changes in physiological uptake could be seen between the three food intake protocols (Figure 1). 

**Figure 2:**
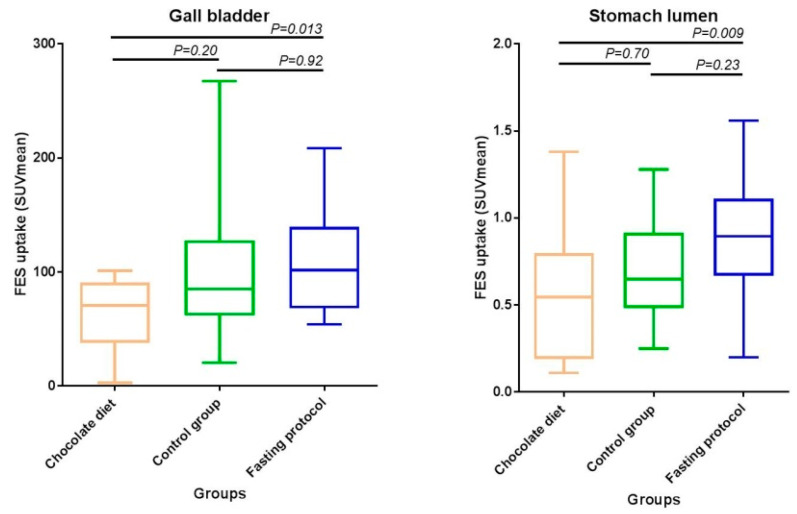
Physiological [^18^F]-FES uptake in gall bladder (**left**) and stomach lumen (**right**) for different food intake protocols.

**Figure 3:**
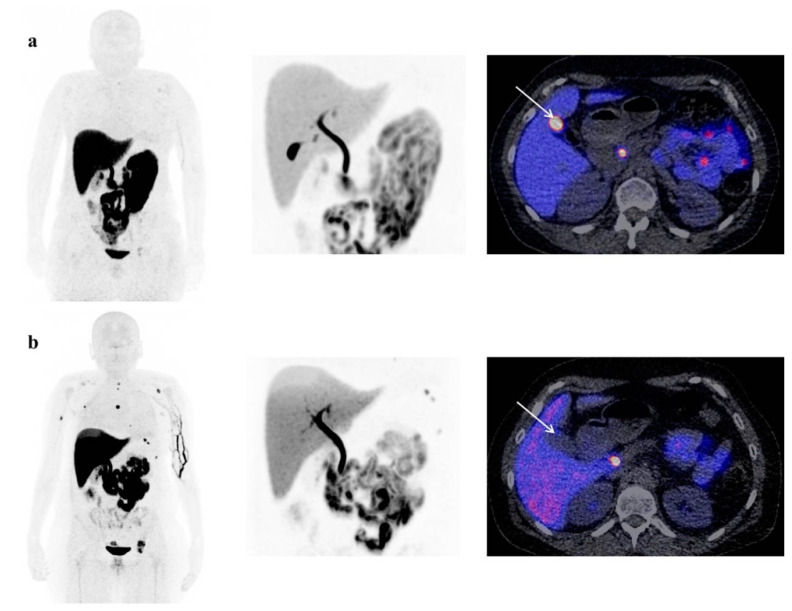
[^18^F]-FES-PET scans of a breast cancer patient showing obvious effects of the chocolate diet. (**a**) A patient without diet restrictions before the scan, with increased physiological uptake in the gall bladder (white arrow). (**b**) The same patient consuming chocolate before the scan, without visible gall bladder uptake (white arrow) due to gall bladder emptying.

Quantitative analysis revealed that physiological [^18^F]-FES uptake in the gall bladder/bile ducts and lumen of the stomach was significantly different between the groups, with the lowest tracer uptake in the chocolate group and highest uptake in the fasting group (see Figure 2). After consumption of chocolate, the median [^18^F]-FES uptake (SUV_mean_) in the gall bladder was 71 (interquartile range 38–91), compared to 85 (62–128) for the control group, and 102 (68–140) for the fasting group (*p* = 0.015). Figure 3 shows the clear visual difference in gall bladder [^18^F]-FES uptake between the chocolate diet and the standard protocol without food restrictions, within the same patient. A post hoc analysis was performed to see which pairs of groups differ significantly in physiological [^18^F]-FES uptake (SUV_mean_) in the gall bladder/bile ducts. The tracer uptake in the gall bladder/bile ducts was significantly lower in patients who ate a chocolate bar compared to patients who were instructed to fast (*p* = 0.013). Comparison of the control and the fasting groups, or the control and the chocolate groups, did not show significant differences in gall bladder uptake (*p* = 0.92 and *p* = 0.20, respectively). Subanalysis of patients whose gall bladder was still in situ (excluding patients with cholecystectomy) showed a similar, but more pronounced, [^18^F]-FES uptake pattern in the gall bladder, with the lowest tracer uptake in the chocolate group and highest uptake in the fasting group (69 (26–87), compared to 85 (62–128), and 108 (78–143), respectively (*p* = 0.007)). A post hoc analysis of differences in gall bladder [^18^F]-FES uptake showed the same statistically significant effect for the chocolate group compared to the fasting group (*p* = 0.005). In a subanalysis of patients without gall bladder (*n* = 7), no significant differences in [^18^F]-FES uptake in the bile ducts between the groups were observed (chocolate diet (*n* = 5): 72 (48–97) versus fasting protocol (*n* = 2): 56 (54–56); *p* = 0.245)).

Physiological [^18^F]-FES uptake in the stomach lumen showed a similar pattern as in the gall bladder (see Figure 2). Tracer uptake in the stomach lumen was also significantly different between the food intake protocols (chocolate diet: 0.55 (0.19–0.80), no diet restrictions: 0.65 (0.48–0.92), and fasting protocol: 0.90 (0.67–1.11); *p* = 0.011)). A post hoc analysis was performed to see which pairs of groups differ significantly in physiological [^18^F]-FES uptake (SUV_mean_) in the stomach lumen. There was a statistically significantly lower [^18^F]-FES uptake in the stomach lumen of patients on the chocolate diet (0.55) than in fasting patients (0.90; *p* = 0.009). The chocolate diet group and control protocol group did not have significantly different tracer uptake in the stomach lumen (*p* = 0.70), and the same applies to the control patients and the patients who had to fast (*p* = 0.23). In addition to the effect in the stomach lumen, there was also a similar trend towards a statistically significant difference in physiological [^18^F]-FES uptake for the stomach wall (*p* = 0.099). 

**Figure 4:**
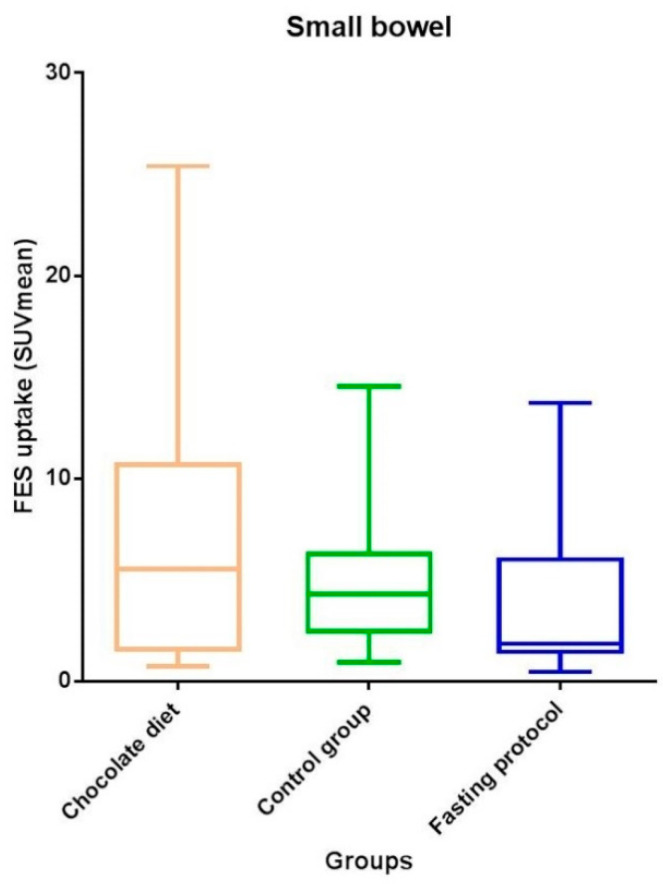
Physiological [^18^F]-FES uptake in small bowel for different food intake protocols.

An inverse relationship regarding physiological [^18^F]-FES uptake was seen for the small bowel, with the lowest median tracer uptake in the fasting group (1.9 (1.3–6.1)) and highest for the chocolate group (5.5 (1.5–10.8); *p* = 0.17; Table 2 and Figure 4)), although the effect of food intake did not reach statistical significance yet. No statistically significant differences in physiological [^18^F]-FES uptake were noted for the other abdominal organs.

## 4. Discussion

In this exploratory study, we assessed the effect of food intake before the [^18^F]-FES-PET scan (a fatty meal (chocolate), fasting, and no diet restrictions) on abdominal background [^18^F]-FES activity in patients with BC.

This is the first study focusing on different patient preparation protocols for [^18^F]-FES-PET studies, with a particular focus on the dietary instructions before imaging. The use of standardized patient preparation protocols for [^18^F]-FES-PET reduces factors that can influence [^18^F]-FES uptake. Given the recent FDA approval of [^18^F]-FES, the optimization and standardization of dietary instructions prior to PET acquisition are highly needed.

Our results showed differences in background [^18^F]-FES activity in the upper abdomen between the food intake protocols. The chocolate group demonstrated significantly decreased [^18^F]-FES uptake in the gall bladder and stomach lumen compared to the fasting group, probably due to a faster excretion from these organs and thereby increased motility through the upper gastrointestinal tract. This result is strengthened by the fact that this effect was only seen in those patients with gall bladder and was not observed in patients with resected gall bladder. The slight decrease in tracer uptake in gall bladder and stomach was indeed accompanied by a nonsignificant increase in tracer uptake in the small bowel. This nonsignificant trend towards a higher uptake in the small bowel after fatty preparation diet can also hamper the detection of tumor in this region. Based on this finding, we cannot conclude that the chocolate diet is superior to the fasting protocol. Further research and additional groups with other patient preparation protocols might possibly help to decrease the tracer uptake in the small bowel as well. One suggestion could be to increase the time interval between tracer injection and the start of the scan. In the present study, all scans were performed 60 min after tracer administration. Scanning at later time points is feasible since we know that [^18^F]-FES uptake in ER-expressing tumors remains stable till at least 120 min after tracer injection [16]. Based on our findings, one could speculate that laxative agents directly after [^18^F]-FES injection and before [^18^F]-FES-PET acquisition can maybe also further reduce the physiological [^18^F]-FES uptake in the upper abdomen, including the small bowel. Different bowel-cleansing methods (including laxatives and dietary restrictions) were investigated in a previous [^18^F]-fluorodeoxyglucose ([^18^F]-FDG)-PET imaging study [17]. They found an increased number of false-positive [^18^F]-FDG-PET scans in the group with laxatives, for example, because of smooth muscle activity. In contrast to [^18^F]-FDG, muscle activity does not affect the physiological [^18^F]-FES uptake. Furthermore, no statistical differences in physiological [^18^F]-FES uptake between the food intake protocols were seen in the other gastrointestinal tract regions, in particular, no trend towards a higher uptake in the large colon. Diffuse uptake of [^18^F]-FES in the colon and the large colon volume may be responsible for this lack of statistical significance. Increasing the time interval may possibly also lead to larger differences in physiological tracer uptake in the lower abdomen. These additional methods (increasing the time interval and using laxatives) can be added to a future clinical prospective trial.

Our quantitative results suggest that a chocolate diet might be helpful in less-disturbing background [^18^F]-FES activity in the gall bladder and stomach. In addition, in one patient we found a visual difference in physiological gall bladder [^18^F]-FES uptake between the chocolate diet and no dietary restriction protocol. This was achieved by stimulating gall bladder contraction with a fatty meal around the time of tracer injection, which would increase the hepatobiliary clearance rate of the [^18^F]-FES tracer. It therefore seems likely that this food intake protocol may increase the contrast between background [^18^F]-FES activity and tracer uptake at tumor sites in the gall bladder and stomach. However, if this finding actually appears in patients with gastrointestinal tract metastasis is still unknown. In the present study, patients with liver metastases were excluded, and none of the patients had metastatic BC to the gastrointestinal tract, except for one patient who had rectal metastases from lobular carcinoma. Therefore, the [^18^F]-FES-PET diagnostic accuracy between the groups needs to be further investigated in patients with ER-positive abdominal metastases, using visual inspection and quantitative analysis. A prospective study, in which the actual diet of the control and fasting groups is included, should assess if the visualization of upper abdominal metastases will improve by using different food intake protocols, resulting in improved detection of ER-positive metastatic cancer. Foremost, it is important to bear in mind that a fasting protocol is less patient-friendly compared to the other two protocols. Ultimately, future studies may be helpful to formulate advice about the best patient preparation instructions.

Gastrointestinal tract involvement is typically found in patients with lobular metastatic BC [18,19]. A previous case report presented [^18^F]-FES-PET findings of a patient with metastatic lobular BC with gastric linitis plastic [20], in which the chocolate protocol may possibly have facilitated tumor detection because of a nonsignificant trend towards lower uptake in the stomach wall. Indeed, metastatic BC to the stomach is rare, and the majority of these patients have ER-positive disease, but ER expression is not only present in BC, but in other (gynecological) tumors as well, such as uterine tumors, ovarian, endometrial, and gastric cancer [4,21,22,23,24,25,26,27,28]. For ovarian cancer, detection of abdominal metastases by [^18^F]-FES-PET could be important. A previous [^18^F]-FES-PET study including ovarian tumors showed that most lesions were intra-abdominal metastases [4]. Detection of these lesions was sometimes hampered by high physiological [^18^F]-FES uptake. 

The main reasons for using chocolate as the fatty meal in our study was its easy availability, as well as the simplicity of its consumption. Furthermore, eating chocolate is also used regularly in the preparation of myocardial perfusion scans and thus can be easily implemented. Milk chocolate was also used in a previous imaging study as a fatty meal to stimulate gall bladder contraction with good results [10]. However, compared to milk chocolate, dark chocolate has higher percentages of fat and may show even more pronounced results.

This study has several limitations, including the retrospective design of the control and fasting groups, and consequently the lack of information about the diet of patients in the control group, as well as the exact number of fast hours in the fasting group. This study did not allow us to clarify whether the effect of chocolate was related to the fat content or caloric value of the meal, so the exact mechanism by which a chocolate diet decreases [^18^F]-FES uptake in stomach and gall bladder could not be clearly defined. The strengths of this study are the sample size of the three groups, the prospective design of the chocolate group, the comparison of multiple food intake protocols, and using matched groups with limited confounding factors that could affect [^18^F]-FES uptake. Furthermore, all scans were performed with standardized acquisition and reconstruction protocols in the same institution. 

In conclusion, in this exploratory study we showed that, compared to fasting, eating chocolate decreases physiological [^18^F]-FES uptake in the gall bladder and stomach. This might be caused by accelerated passage of the tracer. Eating a fatty meal (chocolate) does not significantly decrease [^18^F]-FES uptake further compared to a normal diet. A prospective study, in which patients with abdominal metastases are included, is warranted to confirm this finding.

## Figures and Tables

**Table 1:** Baseline characteristics of breast cancer patients (*n* = 60).

Characteristics	Overall (*n* = 60)	Chocolate (*n* = 20)	Fasting (*n* = 20)	Control (*n* = 20)	*p*-Value
Age, years	68 (30–83)	69 (40–77)	66 (30–74)	69 (40–83)	0.33
Weight, kg	76 ± 15	76 ± 14	77 ± 19	73 ± 12	0.72
Height, cm	167 ± 7	166 ± 8	169 ± 6	166 ± 6	0.50
BMI, kg/m^2^	28 ± 5	28 ± 4	27 ± 6	27 ± 4	0.84
Menopausal status					0.87
Peri/premenopausal	8 (13)	2 (10)	3 (15)	3 (15)	
Postmenopausal	52 (87)	18 (90)	17 (85)	17 (85)	
Cholecystectomy	7 (12)	5 (25)	2 (10)	-	
Treatment [^18^F]-FES-PET					
Aromatase inhibitor	19 (32)	5 (25)	7 (35)	7 (35)	
Chemotherapy	3 (5)	1 (5)	1 (5)	1 (5)	
None	38 (63)	14 (70)	12 (60)	12 (60)	
[^18^F]-FES-PET result ^¶^					
Positive	38 (63)	13 (65)	11 (55)	14 (70)	
Negative	22 (37)	7 (35)	9 (45)	6 (30)	
Stage					
Adjuvant	13 (22)	5 (25)	1 (5)	7 (35)	
Metastatic ^†^	47 (78)	15 (75)	19 (95)	13 (65)	

Values are presented as median (range), mean ± standard deviation (SD), or total number (%). ^¶^ Positive result was defined as clearly visible tumor tracer uptake above background activity; negative result was defined as absence of visual tracer uptake at the location of a metastasis detected by conventional imaging. † Including oligometastases.

**Table 2:** Physiological [^18^F]-FES uptake (SUV_mean_) per food intake protocol and per organ.

Organ	Total	Chocolate	Fasting	Control	*p*-Value
Aortic arch	1.5 (1.2–1.8)	1.3 (1.1–1.7)	1.5 (1.1–1.9)	1.5 (1.3–1.8)	0.65
Thoracic aorta	1.4 (1.2–1.7)	1.3 (1.2–1.6)	1.5 (1.2–1.9)	1.5 (1.2–1.7)	0.36
Liver	12 (9.8–14)	10 (9.4–14)	11 (9.9–15)	13 (11–14)	0.18
Gall bladder or bile ducts	81 (62–109)	71 (38–91)	102 (68–140)	85 (62–128)	0.015
Stomach wall	0.73 (0.62–0.91)	0.67 (0.46–0.88)	0.84 (0.66–1.05)	0.72 (0.60–0.81)	0.099
Stomach lumen	0.70 (0.37–0.93)	0.55 (0.19–0.80)	0.90 (0.67–1.11)	0.65 (0.48–0.92)	0.011
Small bowel	3.9 (1.5–7.1)	5.5 (1.5–10.8)	1.9 (1.3–6.1)	4.3 (2.4–6.4)	0.17
Cecum	0.57 (0.40–1.17)	0.59 (0.46–1.27)	0.49 (0.37–0.66)	0.57 (0.36–2.14)	0.52
Ascending colon	0.54 (0.41–0.74)	0.52 (0.40–0.64)	0.60 (0.39–0.78)	0.62 (0.44–0.78)	0.46
Transverse colon	0.56 (0.41–0.78)	0.52 (0.40–0.72)	0.59 (0.41–0.83)	0.60 (0.41–0.82)	0.68
Descending colon	0.51 (0.38–0.70)	0.57 (0.40–0.64)	0.50 (0.41–0.72)	0.44 (0.28–0.75)	0.63
Sigmoid	0.52 (0.41–0.63)	0.49 (0.39–0.56)	0.52 (0.41–0.65)	0.58 (0.44–0.69)	0.33
Rectum *	0.43 (0.26–0.61)	0.35 (0.25–0.60)	0.48 (0.37–0.64)	0.42 (0.29–0.61)	0.42

Values are presented as median and interquartile range. * In 2 out of 60 patients (one of the chocolate group and one of the control group) it was not possible to measure physiological [^18^F]-FES uptake in the rectum, due to rectal surgery for rectal cancer and rectal metastases from lobular carcinoma.
